# Comparison of Phase Synchronization Measures for Identifying Stimulus-Induced Functional Connectivity in Human Magnetoencephalographic and Simulated Data

**DOI:** 10.3389/fnins.2020.00648

**Published:** 2020-06-19

**Authors:** Kenji Yoshinaga, Masao Matsuhashi, Tatsuya Mima, Hidenao Fukuyama, Ryosuke Takahashi, Takashi Hanakawa, Akio Ikeda

**Affiliations:** ^1^Department of Neurology, Kyoto University Graduate School of Medicine, Kyoto, Japan; ^2^Department of Advanced Neuroimaging, Integrative Brain Imaging Center, National Center of Neurology and Psychiatry, Tokyo, Japan; ^3^Department of Epilepsy, Movement Disorders and Physiology, Kyoto University Graduate School of Medicine, Kyoto, Japan; ^4^Graduate School of Core Ethics and Frontier Sciences, Ritsumeikan University, Kyoto, Japan; ^5^Research and Educational Unit of Leaders for Integrated Medical System, Center for the Promotion of Interdisciplinary Education and Research, Kyoto University, Kyoto, Japan; ^6^Department of Integrated Neuroanatomy and Neuroimaging, Kyoto University Graduate School of Medicine, Kyoto, Japan

**Keywords:** somatosensory system, functional connectivity, phase synchronization, amplitude coherence, cross-periodogram

## Abstract

Phase synchronization measures are widely used for investigating inter-regional functional connectivity (FC) of brain oscillations, but which phase synchronization measure should be chosen for a given experiment remains unclear. Using neuromagnetic brain signals recorded from healthy participants during somatosensory stimuli, we compared the performance of four phase synchronization measures, imaginary part of phase-locking value, imaginary part of coherency (ImCoh), phase lag index and weighted phase lag index (wPLI), for detecting stimulus-induced FCs between the contralateral primary and ipsilateral secondary somatosensory cortices. The analyses revealed that ImCoh exhibited the best performance for detecting stimulus-induced FCs, followed by the wPLI. We found that amplitude weighting, which is related to computing both ImCoh and wPLI, effectively attenuated the influence of noise contamination. A simulation study modeling noise-contaminated periodograms replicated these findings. The present results suggest that the amplitude-dependent measures, ImCoh followed by wPLI, may have the advantage in detecting stimulus-induced FCs.

## Introduction

Functional integration of multiple regions in the central nervous system is an important concept for understanding normal brain function (Ramnani et al., 2004; Stevens, 2009) and the pathophysiology of neuropsychiatric disorders (Stam, 2010; Pettersson-Yeo et al., 2011). Functional integration has been extensively investigated using measures of brain connectivity, and the development of methods for quantifying brain connectivity is a growing field in human neuroscience (Sporns, 2011; Stam and van Straaten, 2012).

Dynamics of brain connectivity are investigated using either functional connectivity (FC), effective connectivity, or both (van Diessen et al., 2015). For computing effective connectivity, researchers have constructed complicated models such as vector autoregression models and state-space models (Bressler and Seth, 2011; Vicente et al., 2011; Friston et al., 2014; Seth et al., 2015), and/or assume directions of causal interaction between regions of interest (Friston et al., 2003, 2013). Hence, although effective connectivity is an indispensable method for determining causality, it requires an *a priori* assumption about the network model. In contrast, FC is simply defined as the statistical dependence of neural signals across spatially remote brain regions (Sporns et al., 2004; Fingelkurts et al., 2005), which can provide prerequisite knowledge for effective connectivity analysis. Because of the simplicity in computing (Bullmore and Sporns, 2009; Bruña et al., 2018), FC analysis is commonly applied to the exploration and exploitation of neuroimaging and electrophysiological data.

Functional connectivity has been measured using several approaches, including phase synchronization measures (Cohen, 2014c), amplitude envelope correlation (Bruns et al., 2000), information theoretical approach (Roulston, 1999) and other methods (Wen et al., 2015; Bakhshayesh et al., 2019) in studies using electroencephalography (EEG) and magnetoencephalography (MEG). Among them, phase synchronization measures, which assess a degree of clustering of phase differences between signals, are widely used (Varela et al., 2001; van Diessen et al., 2015).

Several phase synchronization measures are less susceptible to common source effects (i.e., volume conduction and magnetic field spread). The common source effect is an inevitable and serious problem in electrophysiological studies (Vinck et al., 2011), and cannot be completely eliminated by source reconstruction methods (Schoffelen and Gross, 2009) or solved by independent component analysis (Almeida et al., 2013). The common source effect is problematic in estimating phase synchronization because it can generate spurious phase synchronization with a phase difference of 0 or 180 degrees (zero-lag) among channels or sensors (Cohen, 2014a; Khadem and Hossein-Zadeh, 2014). These phase synchronization measures substantially reduce the common source effects by attenuating zero-lag contributions, and have been more frequently used for investigating FCs in electrophysiological studies (Schoffelen and Gross, 2009).

The phase synchronization measures that are robust to the common source effects include imaginary part of phase-locking value (ImPLV) (Sadaghiani et al., 2012), imaginary part of coherency (ImCoh) (Nolte et al., 2004), phase lag index (PLI) (Stam et al., 2007) and weighted phase lag index (wPLI) (Vinck et al., 2011): ImPLV and PLI are unaffected by amplitudes of signals (i.e., amplitude-independent), while ImCoh and wPLI are affected by amplitudes (i.e., amplitude-dependent). Currently, however, little information is available to help researchers choose an appropriate phase synchronization measure for a specific experimental application. Although some studies compared between FC measures using simulated data (David et al., 2004; Stam et al., 2007; Vinck et al., 2011; Lowet et al., 2016), it is unclear whether these findings can be extrapolated to real human electrophysiological data. In other studies, several FC measures were compared using task-free (resting state) electrophysiological data (Dauwels et al., 2010; Mezeiová and Paluš, 2012; Liang et al., 2016). However, in resting-state EEG/MEG data, it is impossible to differentiate actual FC from false-positive correlations. Other studies compared FC measures from the viewpoint of reproducibility (Colclough et al., 2016; Garcés et al., 2016), but more reproducible FC measures do not necessarily exhibit better performance for detecting FCs. In addition, in many of these studies (Dauwels et al., 2010; Mezeiová and Paluš, 2012; Ard et al., 2015; Colclough et al., 2016; Garcés et al., 2016; Lowet et al., 2016; Bakhshayesh et al., 2019), comparisons were made between different types of FC measures (e.g., amplitude envelope correlation and phase synchronization measures) or between FC measures and effective connectivity measures. However, because different types of FC measures have different functional roles (Mehrkanoon et al., 2014; Guggisberg et al., 2015), they should be regarded as complementary approaches. Based on these considerations, it still remains uncertain which phase synchronization measure is suitable for FC studies in real data.

In the present study, we aimed to compare the four phase synchronization measures, which are less sensitive to near zero-lag interactions (thereby making the four FC measures robust against the common source effects), for identifying functional connectivity in real event-related MEG data. We used neuromagnetic brain signals recorded during exposure to somatosensory stimuli because somatosensory-induced FCs between the primary and secondary somatosensory areas (SI and SII, respectively) have been well documented (Simões et al., 2003; Hagiwara et al., 2010, 2014). We computed FCs between the SI and SII using the four phase synchronization measures, ImPLV, ImCoh, PLI and wPLI, and compared the performance of these measures in identifying somatosensory-induced FCs. We first conducted statistical analysis of somatosensory-induced FCs at an individual level by comparing these FCs with prestimulus (“baseline”) FCs. Second, we compared each of the four FC measures in terms of correct and false detection of stimulus-induced FCs. Third, to examine effects of amplitudes on performance differences among these FC measures, we investigated event-related amplitude changes and cross-periodograms of the time-frequency represented data. Fourth, we analyzed descriptive statistics for these FC measures. Finally, we conducted data simulation for a better understanding of results from the actual MEG data.

## Materials and Methods

### Participants

Thirty-five right-handed healthy volunteers (19 men, 16 women) with a mean age of 23.8 years (from 19 to 30 years) were recruited. All participants were native Japanese speakers and had no history of neuropsychiatric disorders or head trauma. Written informed consent was obtained from all participants. The protocol was approved by the committee of medical ethics at Kyoto University.

### Experimental Task

In the MEG experiment, 0.1-ms electrical pulses were delivered to the digital nerve on each side via ring electrodes. The intensity was adjusted individually so that it was twice the sensory threshold. The inter-stimulus interval varied randomly between 1500 ms and 3500 ms to attenuate the effects of stimulus anticipation of the participants. Three hundred pulses were delivered on each stimulated side. As a result, there were 300 trials on each side of stimulation (hereinafter referred to as the dataset) for each participant. Participants were seated and relaxed with their eyes open during MEG recording. They were asked not to pay attention to the stimuli.

### Data Acquisition

We recorded continuous signals using a whole-head 306 channel MEG system (Elekta Neuromag, Helsinki, Finland) comprised of 102 magnetometers and 204 planar gradiometers. The data were recorded at a sampling rate of 600 Hz with a 0.1 to 200 Hz bandpass filter for removing direct current drifts and signal aliasing. Participants’ head positions relative to the MEG sensor array were measured using four head-position-indicator coils immediately before data recording in each session.

### Data Analysis

We performed the following data analysis with custom-made MATLAB^®^ (MathWorks, Natick, MA, United States) scripts.

#### Preprocessing

The session data were first processed using the signal-space projection method. We then checked inter-session head position movement by computing the Euclidean distance of the head positions in two sessions in each dataset for each participant. Because the inter-session head position distances were less than 7 mm (around one-fifth of the averaged distance between sensors in the MEG system) in all but four of the 63 datasets (7.0, 7.8, 8.7 and 9.0 mm) and the mean of these distances was 3.3 mm, we did not perform head movement correction (Nenonen et al., 2012; Stolk et al., 2013). We used only signals recorded by the 204 gradiometers (hereinafter referred to as channels) for further analyses.

We extracted peri-event signals (0.7 s before to 0.7 s after each stimulus onset) from each trial of these processed data. Trials containing large artifacts (trials in which the maximum peak-to-peak amplitude exceeding 1300 fT/cm) were excluded from further analysis. In this procedure, both datasets of three participants and one dataset of one participant were excluded because a substantial number of trials were rejected due to artifact contamination (50 or more trials out of 300 trials). In the rest of the datasets (63 datasets), the percentages of rejected trials ranged from 0.0% to 14.0% with a mean of 3.1%. After the quality assurance, we concatenated the remaining trials (original trial data, OTD).

#### Selection of Reference Channels

We identified channels over the contralateral SI to the stimulus (c-SI) using somatosensory-evoked fields (SEFs). After the application of a 1-Hz high-pass filter, we computed SEFs by averaging signals of the OTDs. The three channels that clearly showed the early SEF components (i.e., N22m, P30m) on visual inspection (Ishibashi et al., 2000) were selected as reference channels in each dataset (hereinafter referred to as c-SI channels). We here also determined the sensors in which either channel showed SEFs from the SII ipsilateral to the stimulus (i-SII) (channels of these sensors are hereinafter referred to as i-SII channels). The waveforms and latencies of SEFs as well as the location of the sensors over the i-SII were consistent with those reported in previous studies (Kakigi et al., 2000; Wegner et al., 2000).

#### Subtraction of Stimulus-Locked Brain Responses From Signals of Each Trial

When stimuli evoke stimulus-locked responses (e.g., phase resetting) in remote regions, these may result in “apparent” phase locking between those regions even if there is no actual interaction (e.g., common locking to stimuli Simões et al., 2003). When stimulus-locked responses repeatedly occur after somatosensory stimuli, they emerge as somatosensory-evoked potentials/fields (SEPs/SEFs) after trial averaging. Hence, if SEPs/SEFs are present in remote regions, these activities can generate “apparent” FCs. To remove such a spurious effect, a previous study subtracted trial-averaged signals from signals in each trial (Spadone et al., 2015). We applied this method to our MEG data. In practice, we simply subtracted trial-averaged MEG signals (equivalent to SEFs) from each trial data of the OTDs in each individual before computing FCs.

#### Selection of Channel Pairs

We computed the four FC measures (ImCoh, ImPLV, PLI, and wPLI) between each of the c-SI channels and the other 203 channels in each dataset. We transformed the time series of each trial of the OTD into a time-frequency domain using short-time Fourier transform with 333.3 ms (200 time points) Hanning windows with 92.5% overlap. The analyzed frequency range was up to 45 Hz. The FC values were then rescaled in each of the FC measures by dividing them by the root mean squares of the FC values extracted from all the “pixels” (i.e., analysis units of the data represented in the time-frequency space) of all the channels. These rescaled FC values were averaged across the FC measures in each pixel and channel.

Using this rescaled and averaged FC values, we selected the channel pair (one for the c-SI channels and the other for the i-SII channels) between which the greatest FC values were identified during the poststimulus period (0–0.5 s) in each dataset. In each of the datasets where somatosensory-induced FCs were poorly identified visually (19 out of 63 datasets), we selected the channel pair, which showed the largest SEFs in the c-SI and the i-SII channels.

#### Constructing Surrogate Data

To focus on stimulus-induced FCs, we needed to compare poststimulus FCs with resting-state (“baseline”) FCs (Delorme and Makeig, 2004). In addition, we used the cluster-size-based test to solve the multiple comparison problem in statistical analysis for our time-frequency represented data (Maris and Oostenveld, 2007). Because there is no *a priori* knowledge about null distributions of these test statistics (i.e., resting-state FC values and cluster sizes), we used a surrogate data method to obtain these null distributions (Lachaux et al., 1999).

**FIGURE 1:**
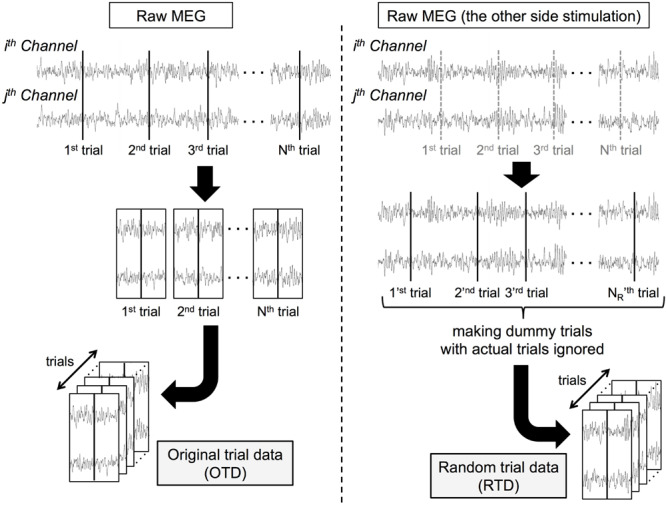
Processing pipeline for constructing surrogate data. Each “trial K” and “trial K_*R*_” represents Kth actual trial onset (K  =  1,2,  … ,  N) and K_*R*_ th dummy trial onset (K_*R*_   =  1,2,  … ,  N_*R*_). c-SI Ch: one of a channel pair over the primary somatosensory cortex contralateral to the stimuli, i-SII Ch: the other of a channel pair over the secondary somatosensory cortex ipsilateral to the stimuli.

We generated surrogate data of each dataset in the following procedure (Figure 1). From the same participant’s MEG data recorded when the stimuli were delivered on the other side, we randomly selected 1,100 dummy trial onsets. After trial rejection as in section “MATERIALS AND METHODS Preprocessing,” we obtained surrogate trial data by extracting peri-event signals (random trial data, RTD). We generated the RTDs in each dataset (the number of trials ranged from 853 to 1,073 among all the datasets). In these RTDs, although the timing of stimulus events was lost, the temporal relationship of the signal time series was preserved across all channels. Note that the RTD had the same statistical properties as resting-state (and prestimulus) MEG data and served as control data for stimulus-induced phase synchronization.

#### Performance Analysis of Functional Connectivity Measures

##### Statistical analysis for identifying significant functional connectivities

For assessing the significance of somatosensory-induced FCs, we used the following two-step thresholding method. We first conducted value-based thresholding in each pixel of the time-frequency space (pixel-based test). We then conducted cluster-size-based thresholding by grouping neighboring pixels with suprathreshold values as clusters (cluster-based test) (Cohen, 2014a) for solving the multiple comparison problem. To infer significance for these tests, we computed histograms approximating probability distributions of these statistics using the generated surrogate data. Because cluster sizes took integers greater than or equal to 0, we were able to evaluate the statistical significance of clusters in a more precise manner by computing cluster sizes at a finer scale. To this end, we analyzed the data with relatively small time bins (10 ms), resulting in a 97% overlapping Hanning window. Note that this rescaling of time bins does not change the order of cluster sizes.

**FIGURE 2:**
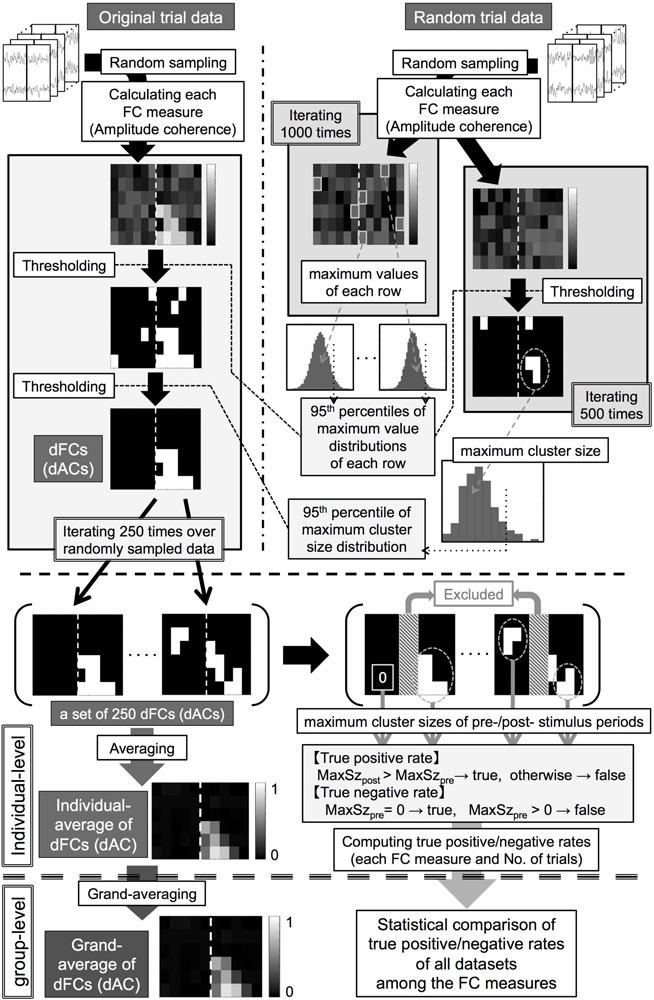
(Upper panel, thick dashed line) Processing pipeline of statistical analysis for identifying significant functional connectivities (FCs) and amplitude coherences (ACs). (Middle panel, thick dashed line) Processing pipeline for an individual-level analysis for obtaining the individual-averages of double-thresholded functional connectivities and true positive/negative rates. (Lower panel, thick dashed line) Processing pipeline for a group-level analysis for the individual results. dFCs/dACs: double-thresholded FCs/ACs, MaxSz_*pre*_/MaxSz_*post*_: maximum cluster sizes of the prestimulus/poststimulus periods.

We obtained the thresholds for the pixel-based test in each dataset as follows (Figure 2, upper right).

(1) Computing each FC measure between the pre-selected channel pair using *N* trials of data randomly sampled from the RTD (*N* = 100, 200) and collecting the maximum value in each frequency row 1000 times to compute null distributions of surrogate FC values in each frequency row and each of the FC measures.
(2) Computing the 95th percentiles of these null distributions as threshold values of each frequency row and each of the FC measures for the pixel-based test.
  We obtained the thresholds for the cluster-based test in each dataset as follows (Figure 2, upper right).
(3) Computing each of the FC measures between the pre-selected channel pair using *N* trials of data randomly sampled from the RTD (*N* = 100, 200) and thresholding these surrogate FC values by applying the corresponding threshold values computed in the step (2) in each frequency row and each FC measure.
(4) Grouping 4-connected neighboring pixels (constituted with time bins and frequency bins) with suprathreshold values as clusters and collecting the maximum cluster size (number of pixels in each cluster) in each FC measure.
(5) Iterating steps (3) + (4) 500 times to obtain null distributions of the maximum cluster size in each of the FC measures and computing the 95th percentiles of these null distributions as threshold cluster sizes of each FC measure for the cluster-based test.
  We then applied the two-step thresholding method to each OTD as follows (Figure 2, upper left).
(6) Computing each FC measure between the pre-selected channel pair using *N* trials (*N* = 100, 200) randomly sampled from the OTD.
(7) Conducting the pixel-based test to these FC values in the same way to the step (3) and grouping 4-connected neighboring pixels with suprathreshold values as clusters.
(8) Leaving clusters with cluster sizes larger than the threshold cluster size as statistically significant FCs in each FC measure (double-thresholded FCs, dFCs).
(9) Iterating steps (6) + (7) + (8) 250 times.

We thus obtained sets of 250 dFCs for each number of trials and each FC measure in each dataset. This procedure controlled for a family-wise error rate of each dFC at 0.05 after the multiple comparison correction in the time-frequency space.

We averaged 250 dFCs to obtain the individual-level averages of dFCs in each dataset. We then computed the grand-averages of dFCs by averaging these individual-averages of dFCs across all the datasets for each number of trials and each of the FC measures.

##### Comparison of performance among the functional connectivity measures

Using the RTDs as the surrogate data, by construction, we should be able to detect stimulus-induced FCs only during the poststimulus period but not during the prestimulus period (at a 0.05 family-wise error rate). Thus, to assess correct and false detection rates of these FC measures, we computed true positive rates (TPRs) and true negative rates (TNRs) using the dFCs in the following way. First, time bins in each 50-ms period immediately before and after the stimulus onset were excluded from this analysis because time windows of these bins were considerably affected by both prestimulus and poststimulus brain signals. We then computed the maximum cluster size in each of the prestimulus and poststimulus periods for each dFC (zero if no cluster survived). We computed the TPRs as proportions of dFCs in which the maximum cluster sizes in the poststimulus period were greater than those in the prestimulus period in each set of 250 dFCs. We computed the TNRs as proportions of dFCs in which the maximum cluster size in the prestimulus period was greater than zero among each set of 250 dFCs. We thus obtained the TPRs and TNRs for each number of trials and each FC measure in each dataset.

To detect significant differences in these TPRs and TNRs among the FC measures, we compared these rates between the FC measures for each number of trials (i.e., 100 and 200) using the Friedman test. A *post hoc* analysis was conducted using the one-tailed Wilcoxon signed-rank test to the directional alternative hypothesis, which states that the rates of one FC measure were greater than those of another measure (4 × 3 pairs in total). For the multiple comparison procedure, the Benjamini–Hochberg method (Benjamini and Hochberg, 1995) was used to control for the false discovery rate below 0.05.

#### Amplitude Effect Analysis

Amplitudes of time-frequency transformed signals can affect FC values, particularly when computing coherence and related measures (e.g., ImCoh and wPLI). As shown in Appendix, amplitudes work as a weighting factor of cross-periodograms for computing these measures (i.e., amplitude weighting). To investigate the effects of signal amplitudes on performance differences among the FC measures, we conducted the following analyses.

##### Amplitude coherence analysis

To investigate event-related amplitude changes, we computed event-related amplitude coherence (AC) in each dataset according to the formula in Appendix (Srinath and Ray, 2014). For statistical analysis, we applied the same procedure with the FC measure analysis (see section “MATERIALS AND METHODS Statistical analysis for identifying significant functional connectivities” and Figure 2) using the RTDs as the surrogate data. The number of trials for random sampling was fixed to 200 in this analysis. We computed a set of 250 double-thresholded ACs (dACs, both value- and size-based thresholded) in each dataset. We then computed the grand-average of dACs by averaging these sets of 250 dACs over all the datasets.

##### Cross-periodogram analysis

To investigate the relationship of the amplitudes of signals with the synchronized phases, we analyzed cross-periodograms (complex values) from pixels of the time-frequency represented OTD. Computing the rescaled and averaged FCs as in section “MATERIALS AND METHODS Selection of Channel Pairs,” we extracted cross-periodograms from the pixel with the greatest value in each dataset, and computed the magnitudes (i.e., amplitudes) and angles (i.e., phases) of these cross-periodograms.

We computed phase lags (angles of phase locking) of stimulus-induced FCs in each dataset as the mean angle of angles of complex-valued phase-locking values and coherencies computed using the cross-periodograms. The phases of the cross-periodograms were converted to the angles relative to the estimated phase lag in each dataset (relative phases ranging from 0 to pi). We then summarized the relationship between the amplitudes of the cross-periodograms and the relative phases derived from all datasets by dividing the amplitudes into 9 bins from 0 to pi according to the relative phases.

We also estimated signal-to-noise ratios (SNRs) of the stimulus-induced FCs using these cross-periodograms because the phase lags and SNRs were likely the major determining factors for the performance of the FC measures. The SNR was computed in each dataset as the ratio of the squared magnitude of mean of the cross-periodograms and the squared magnitudes of the residuals (after subtracting the mean from the cross-periodograms). Note that this computation was based on the assumption that phase-locked cross-periodograms were constant across trials. When phase-locked cross-periodograms were not constant (i.e., trial-by-trial variations), these variations were included in the residuals, which probably resulted in an underestimation of the SNRs.

#### Descriptive Statistics of Functional Connectivity Measures

To investigate the underlying mechanism for the performance difference between the two amplitude-dependent measures (ImCoh and wPLI) (see section “RESULTS Comparison of Performance Across Functional Connectivity Measures”), we computed a range of population parameters as means and coefficients of variance (CVs) of these two FC measures in each pixel in each dataset. We first defined a mask image for stimulus-induced FCs in the time-frequency space by thresholding the grand-averaged dFCs. We also defined the pixels, ranging from 20 to 40 Hz in the frequency bands and 0.5 to 0.3 s before the stimulus onset in the time bins, as a mask image for prestimulus FCs. Using a bootstrap method (number of trials = 200), we obtained sets of FC values from these two mask images in the two FC measures. To correct for the difference in scales of the FC values between the FC measures, we divided these sets of FC values by the averaged prestimulus FC value in each FC measure, producing normalized FC values. We then computed means and CVs of these sets of normalized FC values, and compared these values between the FC measures. A detail of this analysis is described in Supplementary Material 1 and Supplementary Figure S1.

#### Data Simulation of Noise-Contaminated Periodograms

To substantiate the preceding results based on the actual MEG data from a numerical point of view, we applied the same analyses to simulated noise-contaminated periodograms. We simply generated a single pair or a set of 10 pairs (multiple pairs) of phase-locked complex numbers (signals) and added random complex numbers (noises) to them. For this data simulation, we set three parameters: SNRs, phase lags and numbers of trials. We used a surrogate data method for statistical analysis in this data simulation. As surrogate simulation data, we generated a single pair or multiple pairs of phase-UNLOCKED complex numbers with noise. Using the thresholded results, we computed true positive rates (TPRs) as the proportions of the simulations where one or more simulated FCs survived. We then compared TPRs among the FC measures. We also conducted the descriptive statistics analysis for ImCoh and wPLI using these simulated data (number of trials = 200). Using the FC values in the single-pair simulation, we computed means and CVs of these FC values normalized by the FC values computed from the surrogate simulation data. A detail of the data simulation is described in Supplementary Material 2.

## Results

In all 63 datasets, SEFs were visually identified in the channels over the c-SI and i-SII. After selecting a channel pair in each dataset (see section “MATERIALS AND METHODS Section Selection of Channel Pairs”), we conducted the following analyses.

### Comparison of Performance Across Functional Connectivity Measures

**FIGURE 3:**
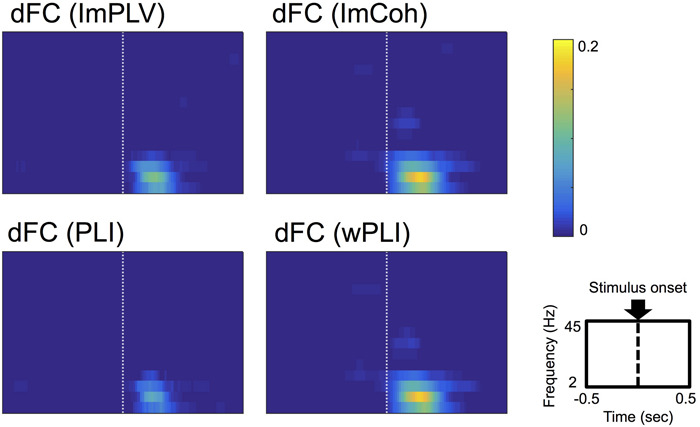
Grand-averages of double-thresholded functional connectivities (dFCs) across all the datasets. As shown in the rightmost-bottom panel, each panel shows data in the time range (abscissa) of 0.5 s before to 0.5 s after the stimulus onset (0 s) and in the frequency range (ordinate) between 2 Hz and 45 Hz. Note that each pixel value represents a proportion of the corresponding pixel to be in significant clusters among all 250 sets of random sampling and all datasets.

Conspicuous stimulus-induced FCs were found during a period of 50–250 ms after the stimulus and in the frequencies ranging from 3 Hz to 12 Hz in all FC measures (Figure 3). Both ImCoh and wPLI showed larger and temporally smoother stimulus-induced FCs than ImPLV and PLI. Only in ImCoh and wPLI, stimulus-induced FCs were also found in the 18–24 Hz frequencies, but these FCs were less obvious and lasted for a shorter time than the stimulus-induced FCs in the 3–12 Hz frequencies.

**FIGURE 4:**
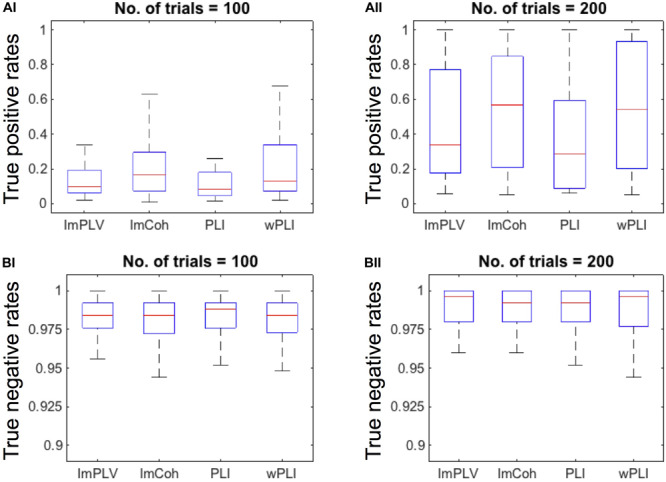
**(A)** Box plots of the true positive rates (TPRs) with the number of trials being 100 **(AI)** and 200 **(AII)**. For this plotting purpose, we only used the datasets (*n* = 35) in which the TPRs for the number of trials 200 were above 0.05 across all the FC measures. **(B)** Box plots of the true negative rates (TNRs) of all the datasets when the number of trials is100 **(BI)** and 200 **(BII)**. On each box plot, the red line, the bottom and top edges of the box represent the median, 25th and 75th percentiles of the TPRs, respectively. The whiskers represent the most extreme values of the TPRs except outliers.

The TPR analysis revealed differences in the TPRs among the FC measures (Figure 4A). A Friedman test revealed significant differences in the TPRs among the FC measures regardless of the numbers of trials (*p* < 0.001 for both 100 trials and 200 trials). In the *post hoc* analysis (Table 1), the TPRs of ImCoh and wPLI were significantly greater than those of ImPLV and PLI regardless of the number of trials (false discovery rate, *p* < 0.05). The TPRs of ImCoh were significantly greater than those of wPLI for 100 trials, while not for 200 trials.

**TABLE 1:** Results of the *post hoc* analysis of differences in the true positive rates between each pair of the functional connectivity measures when the number of trials was 100 **(A)** and 200 **(B)**.

**(A)**					
		**Inferior**			
		**ImPLV**	**ImCoh**	**PLI**	**wPLI**
Superior	ImPLV	N/A	1.000	0.000*	0.999
	ImCoh	0.000*	N/A	0.000*	0.015*
	PLI	1.000	1.000	N/A	1.000
	wPLI	0.001*	0.985	0.000*	N/A
**(B)**					
Superior	ImPLV	N/A	0.999	0.000*	0.999
	ImCoh	0.001*	N/A	0.000*	0.199
	PLI	1.000	1.000	N/A	1.000
	wPLI	0.001*	0.804	0.000*	N/A

Each value represents *p*-value of the one-tailed Wilcoxon signed-rank test under the null hypothesis that there is no difference in the rates between each pair of the measures of the row header and the column header. N/A, not applicable. *False discovery rate < 0.05.

Contrary to the TPRs, the TNRs were around 0.975 for all the FC measures and for both the numbers of trials (Figure 4B). The Friedman test revealed no statistically significant differences among the FC measures for either 100 (*p* = 0.934) or 200 (*p* = 0.863) trials. Thus, the *post hoc* analysis was not conducted for the TNRs.

### Amplitude Effect Analysis

**FIGURE 5:**
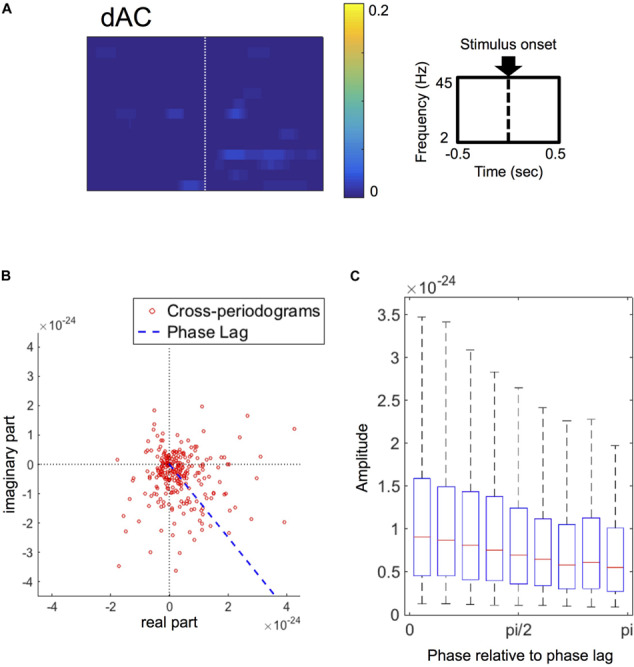
**(A)** Grand-average of double-thresholded amplitude coherences (dACs) in the time range (abscissa) of 0.5 s before to 0.5 s after the stimulus onset (0 s) and in the frequency range (ordinate) between 2 Hz and 45 Hz. Note that each pixel value represents a proportion of the corresponding pixel to be in significant clusters among all 250 sets of random sampling and all datasets. **(B)** Polar plot of the cross-periodograms of one representative dataset (dataset 42). Each dot represents each cross-periodogram and the blue dashed line represents the direction of the estimated phase lag. The distribution of the plot appear shifted from the origin toward the direction at the estimated phase lag. **(C)** Box plots of amplitudes of cross-periodograms sorted into 9 bins according to these phases relative to angles of phase locking (i.e., phase lags). These 9 bins are successive bins from 0 to pi. On each box plot, the red line, the bottom and top edges of the box represent the median, 25th and 75th percentiles of the amplitudes in each bin, respectively. The whiskers represent the 10th and 90th percentiles.

The grand-average of dACs was less evident (Figure 5A) in comparison with the grand-averages of dFCs (see Figure 3), suggesting that ACs were not consistent in the present dataset. Only a few significant clusters were identified in the grand-averages of dACs, which showed little or no overlap with the clusters identified in the grand-averages of dFCs. Based on this result, we considered that the event-related amplitude changes contributed little to the results of the FC performance analysis.

Focusing on the amplitudes of cross-periodograms, the amplitudes became greater as these phases became closer to the estimated phase lag as exemplified in Figure 5B. This finding was evident in the group level analysis, where the amplitudes gradually decreased as the relative phases became larger (Figure 5C). The amplitudes were significantly different among these bins (*p* < 0.001, Kruskal–Wallis test). These findings indicate that cross-periodograms with angles close to the actual phase lag have large amplitudes and thus should be heavily weighted in the amplitude weighting for computing wPLI and ImCoh.

The phase lags of the stimulus-induced FCs, which were rescaled from 0 to pi/2 as the minimum angle from the horizontal in the polar coordinates, ranged from 0.300 to 0.997 times of pi/2 among all datasets (25th–75th percentiles: 0.544–0.846 times of pi/2), and were more than pi/4 in 50 out of these 63 datasets. The SNRs ranged from 0.012 to 0.207 (25th–75th percentiles: 0.026–0.069).

### Comparison of Descriptive Statistics Between Amplitude-Dependent FC Measures

**FIGURE 6:**
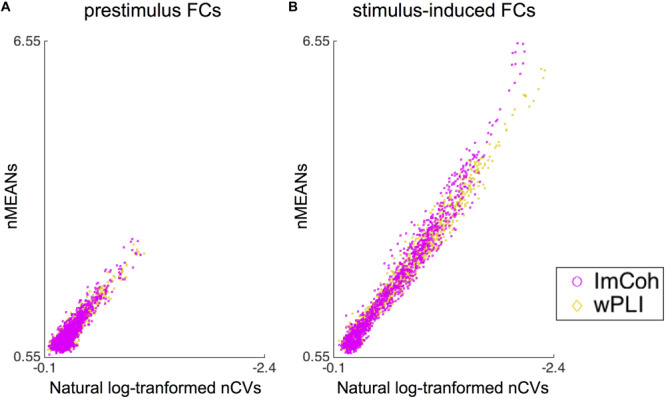
Results of the descriptive statistics analysis. **(A)** Dot plots of means against natural log-transformed coefficients of variance of normalized functional connectivity (FC) values retrieved from the prestimulus FC mask. The plots are well overlapping between ImCoh and wPLI. **(B)** Dot plots of means against natural log-transformed coefficients of variance of normalized FC values retrieved from the stimulus-induced FC mask. The dot plot of ImCoh appears gradually shifted upward and leftward in comparison with that of wPLI as means of normalized FC values become greater. Note that values increase from right to left in the *x*-axis of both plots. nMEANs, means of normalized FC values; nCVs, coefficients of variance of normalized FC values.

The descriptive statistics analysis revealed that statistical properties (i.e., means and CVs) of normalized FCs were somewhat different between ImCoh and wPLI in stimulus-induced FCs, while quite similar in prestimulus FCs (Figure 6). It appeared that both the means and CVs of normalized FCs were larger in ImCoh than those in wPLI. These differences between ImCoh and wPLI were more evident when the means of normalized FCs were high. For statistical confirmation of this observation, we conducted a linear regression analysis between means (response variable) and CVs (explanatory variable) of the normalized stimulus-induced FCs. The estimated slope and intercept were −2.582 and 0.077, respectively, for ImCoh, and −2.382 and 0.183, respectively, for wPLI. The estimated slopes were significantly different between ImCoh and wPLI (*p* < 0.001, *F*-test). These findings indicate that ImCoh take higher mean and CVs than wPLI in pixels where the stimulus-induced FCs are stronger.

### Data Simulation of Noise-Contaminated Periodograms

**FIGURE 7:**
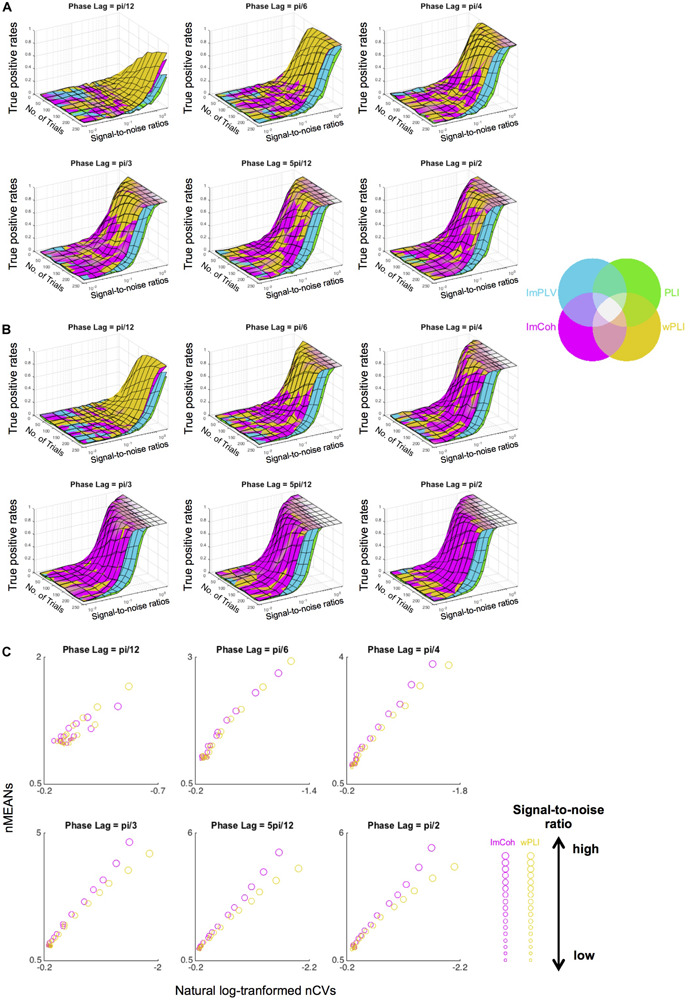
Results of the data simulation of periodograms. **(A,B)** Surface plots of the true positive rates against numbers of trials and signal-to-noise ratios (SNRs) at each angle in the single-pair simulation **(A)** and multiple-pair simulation **(B)**. Surfaces of the plots of each functional connectivity (FC) measure colored with the corresponding color in the color map are overlaid as layers so that the measure with the greatest rate is identified in the uppermost layer. Overall, ImCoh and wPLI show higher rates in most situations. ImCoh tends to have higher rates in the multiple-pair simulation or at larger phase lags, whereas wPLI tends to have higher rates in the single-pair simulation or at smaller phase lags. **(C)** Dot plots of the means against the coefficient of variances of the normalized FCs in the descriptive statistics analysis for the simulated data. The sizes of dots change depending on the SNRs (getting larger as the SNRs become higher). Note that values increase from right to left in the x-axis of all plots. nMEANs, means of normalized FCs; nCVs, coefficients of variance of normalized FCs.

The simulation study supported the results from the actual MEG data, revealing that ImCoh and wPLI showed higher TPRs than ImPLV and PLI in almost all pairs of parameters. The results were roughly consistent across the single-pair (Figure 7A) and the multiple-pair simulation (Figure 7B). ImCoh showed slightly higher TPRs than wPLI when the phase lags were relatively large (equal to or more than pi/4). This pattern was more evident in the case of the multiple-pair simulation than the single-pair simulation. On the contrary, wPLI showed higher TPRs than ImCoh when the phase lags were small (i.e., pi/12). ImCoh showed slightly higher TPRs than wPLI when the SNR was low and vice versa at some of the phase lags (e.g., pi/6, pi/4) in the single-pair simulation, but this pattern was indistinct in the multiple-pair simulation. The number of trials appeared to be less influential on the differences of TPRs than the phase lags. Note that the range of the SNRs (from 0.009 to 2.081) in the simulation study covered that of our real MEG data (see section “RESULTS Amplitude Effect Analysis”).

The properties of descriptive statistics (means and CVs) showed similar results to those of the actual MEG data (Figure 7C), particularly at phase lags equal to or greater than pi/4. That is, both the means and CVs of normalized FC values were greater in ImCoh than those in wPLI as the means became greater. This result supported an interpretation that the tendency for larger mean values at the expense of larger CVs related to the slightly better performance of ImCoh over wPLI.

## Discussion

In the present study, we compared the performance of the four FC measures (all of which are robust to the effects of common sources) for detecting somatosensory stimulus-induced FCs. ImCoh and wPLI exhibited superior performance in detecting the stimulus-induced FCs compared with ImPLV and PLI while the four methods, similarly, rejected prestimulus FCs to be taken as stimulus-induced FCs. The comparison between ImCoh and wPLI revealed that ImCoh showed slightly superior performance in detecting the stimulus-induced FCs.

### Somatosensory-Induced Functional Connectivity Between the Primary and Secondary Somatosensory Areas

In the present study, we focused on somatosensory stimulus-induced FCs between the c-SI and i-SII (Kakigi et al., 2000; Schnitzler et al., 2000; Lin and Forss, 2002; Kaas, 2012). Previous studies identified somatosensory-induced phase synchronization between these areas in humans (Simões et al., 2003; Blatow et al., 2007; Hagiwara et al., 2010, 2014; Weisz et al., 2014), and some of them reported non-zero-lag phase synchronization (Hagiwara et al., 2014; Weisz et al., 2014). These FCs are thought to be supported by fiber connections between these areas in both humans and non-human primates (Cipolloni and Pandya, 1999; Disbrow et al., 2003; Eickhoff et al., 2010). Based on these previous findings, we consider that the present MEG data should contain somatosensory stimulus-induced FCs between the c-SI and i-SII as a conceivable example of stimulus-induced FCs. It remained unclear, however, as to which measure to be chosen to detect the should-be-present FC. This problem motivated us to run the present study.

The somatosensory-induced FCs in the present study showed smaller FCs in a relatively high frequency range (e.g., beta to low gamma bands) in reference to previous studies (Simões et al., 2003; Hagiwara et al., 2010, 2014). Some experimental and technical differences may explain this discrepancy. First, the stimulation method we used was slightly different from that employed in the previous studies. While electrical stimuli were delivered to the median nerve at the wrist in these previous studies, we stimulated the digit nerve of the thumb. Because the median nerve includes motor branches and sensory branches other than the digit nerve of the thumb, the median nerve stimulation was expected to induce stronger inputs than the digit nerve stimulation. Second, the FC measures are different. We focused on the FC measures that are robust to the effects of common sources. According to these definitions, these measures are assumed to be less sensitive than other FC measures such as phase-locking value or coherence (Stam et al., 2007; Vinck et al., 2011), although this sacrificing sensitivity is essential for achieving the robustness to the common source effects. Third, for statistical analysis, we used a cluster-size-based thresholding, which could potentially exhibit a bias toward detecting FCs with lower frequencies. This is because low-frequency FCs are generally smoother in the time domain than high-frequency FCs (Cohen, 2014b). Hence, cluster sizes tend to be larger at low frequencies than high frequencies, resulting in a tendency for FCs of lower frequencies to survive in cluster-based analysis.

### Validation of the Two-Step Thresholding Procedure Using a Surrogate Data Method

When we applied the two-step statistical procedure to the “baseline” data (i.e., presimulus or resting-state FCs), no cluster was expected to survive in 95% of cases if the procedure correctly controlled for a family-wise error rate of 0.05. The results of the TNR analysis revealed that TNRs were around 0.975 irrespective of the FC measures and the number of trials, yielding the family-wise error rates of the two-step procedure around 0.025. Although we controlled the family-wise error rates of false detection of any clusters only during the peristimulus periods (prestimulus and poststimulus) under 0.05, we considered clusters only during the prestimulus period for the TNR analysis, which explains why the family-wise error rates were approximately half of 0.05 in the current result. For this reason, we confirmed that our procedure is a valid method to control the family-wise error rate at 0.05 for statistical analysis of time-frequency represented data.

### Comparison of Performance Between the Amplitude-Independent and Dependent Measures

ImCoh and wPLI showed significantly higher TPR than ImPLV and PLI, while there was no significant difference in TNRs among the four FC measures. These results suggest that ImCoh and wPLI had advantages in detecting somatosensory-induced functional connectivities over ImPLV and PLI, at least in the experimental condition employed here. Both ImCoh and wPLI are computed from coherences, while ImPLV and PLI are computed from phase information. As noted in several previous studies (Lachaux et al., 2002; Srinath and Ray, 2014), coherences can be regarded as amplitude-weighted phase-locking values. Therefore, the differences between ImCoh/wPLI and ImPLV/PLI are presumed to arise from the effects of amplitude weighting.

We first investigated the possibility that event-related changes in amplitude of oscillations caused the difference in the performance. However, the AC analysis revealed that event-related amplitude changes were not significant and had little effect on the difference of dFCs among the four FC measures in the present data. This finding indicates that event-related amplitude changes were less likely to explain the difference between ImCoh/wPLI and ImPLV/PLI.

We next examined cross-periodograms because phase synchronization is computed using this information (Gordon et al., 2013). This cross-periodogram analysis showed that, by considering the amplitudes, cross-periodograms with angles closer to the proper angle were more heavily weighted (see Figure 5C). Because of this amplitude weighting, the amplitude-dependent measures are expected to show better performance than the amplitude-independent measures in detecting FCs between correlated signals contaminated by noises. Note that this amplitude weighting does not depend on the existence of event-related amplitude coherences.

### Comparison of Performance Between the Amplitude-Dependent Measures

Between the two amplitude-dependent FC measures, ImCoh exhibited slightly better performance than wPLI in the TPR analysis (Figure 4 and Table 1). This difference can be explained through our descriptive statistics analysis. In general, both higher contrast of means between two groups and lower CVs (or standard deviations) in each group are expected to increase the probability of detecting a significant difference between these groups. There seemed to be a trade-off statistical property between means and variances in ImCoh (i.e., higher means and higher CVs of the normalized FC values in comparison with those in wPLI), judging from the distribution of the CVs against the means of the normalized FC values (see Figure 6B). Our data simulation replicated this finding, particularly when angles of cross-periodograms were relatively large (Figure 7C). Considering the results of our TPR analysis, the greater mean values seemed to be more beneficial than the lower CVs in this trade-off relationship.

Our data simulation also revealed that ImCoh showed better performance in considering periodograms of multiple-pair data than those of single-pair data. Because considering multiple-pair data is expected to reduce the influence of CVs in comparison with single-pair data, it can be inferred that the difference of TRPs was more related to the difference of means in our descriptive statistics analysis. Therefore, the higher contrast of means in ImCoh was likely to be more crucial for the statistical significance of stimulus-induced FCs in the time-frequency space.

### Comparison With Previous Studies

Only a few previous studies directly compared FC detection performance among FC measures that are not affected by the common source effects (Stam et al., 2007; Vinck et al., 2011). These studies reported an advantage for PLI/wPLI over ImCoh in contrast to our study. There are several possible reasons for this discrepancy. First, as also noted in the previous studies (Stam et al., 2007; Vinck et al., 2011), phase lags of FCs are thought to affect the performance of these FC measures. In our real MEG data, most of the stimulus-induced FCs had phase lags larger than pi/4, where ImCoh showed better performance in both the actual MEG data and the simulated data. On the contrary, our data simulation revealed that wPLI showed higher TPRs when the phase lags were close to zero (e.g., phase lag = pi/12 in Figures 7A,B). To investigate the relationship of phase lags to the performance of these FC measures in actual brain signals, further studies should be conducted with other data.

Second, PLI and wPLI are thought to be robust to noise contamination when noise is small enough not to change the signs of imaginary parts of cross-periodograms. In simulated data and human EEG data of absence seizures in a previous report (Stam et al., 2007), PLI and wPLI showed better performance in detecting FCs than ImCoh. However, in our cross-periodogram analysis (Figure 5B), the dot plots of cross-periodograms were widely distributed across the real axis in the polar coordinates. This finding suggests that actual brain signals are not so stable that signs of imaginary parts of cross-periodograms stay the same. Thus, we presumed that these two factors (phase lag of FCs and level of noise) would explain the discrepancy between our results and the previous reports.

### Limitations of the Present Study

The current results focusing on the stimulus-induced FC are not directly applicable to resting-state electrophysiological data. However, our findings suggest that the amplitude weighting, which can attenuate the effect of noise on computing FCs, may be critical for the difference of performance between the amplitude-dependent and independent measures. Because the amplitude weighting is presumed to be effective in computing resting-state FCs, our results may be applicable, to some extent, to resting-state FC analysis.

An important issue of cluster-based analysis should be considered when interpreting the current results. In the cluster-based method, we considered clusters composed of time and frequency bins in the time-frequency space. However, to use the sizes of these clusters as a fair measure of extent, the assumption must be satisfied that frequency bins are equivalent to temporal bins in terms of smoothness (i.e., a degree of correlation between adjacent bins). However, this may not be the case because, in time-frequency transform methods such as short-time Fourier transform, the smoothness of frequency bins usually differs from that of temporal bins (Cohen, 2014b). Using different temporal and frequency bins in time-frequency transform can lead to differences in threshold values in cluster-based analysis, resulting in differences in the results of statistical analysis, even when conducted with the same data. To date, there is no validated solution for this problem. Thus, further improvements are required to address this limitation of cluster-based methods.

## Conclusion

In the present study, focusing on the FC measures that are robust to the common source effects, we investigated the performance of these measures in detecting somatosensory-induced FCs. Overall, ImCoh was the most sensitive method for detecting stimulus-induced FCs, at least in the present experimental condition. The difference in the performance was partially explained by the notion that the amplitude weighting was effective for attenuating negative effects of noise contamination in computing FCs and by different properties of descriptive statistics among these measures. The present work offers a useful insight into functional connectivity studies with electrophysiological data.

## Data Availability Statement

The datasets generated for this study are available on request to the corresponding author.

## Ethics Statement

The studies involving human participants were reviewed and approved by the committee of medical ethics at Kyoto University. The patients/participants provided their written informed consent to participate in this study.

## Author Contributions

KY, MM, HF, RT, and TH did the proposal of the work and experimental design. KY acquired the data. KY and MM performed the data analysis including scripting. KY, MM, TM, TH, and AI wrote and revised the manuscript. All of the authors have read and approved the manuscript.

## Conflict of Interest

Department of Epilepsy, Movement Disorders and Physiology (AI and MM) is the Industry-Academia Collaboration Courses, supported by Eisai Co., Ltd., Nihon Kohden Corporation, Otsuka Pharmaceuticals Co., and UCB Japan Co., Ltd.

The remaining authors declare that the research was conducted in the absence of any commercial or financial relationships that could be construed as a potential conflict of interest.

## Supplementary Material

FIGURE S1 Processing pipeline for investigating descriptive statistics of the functional connectivity measures. The mask images for stimulus-induced FCs and for prestimulus FCs were defined using the grand-averages of double-thresholded FCs (dFCs). FC, functional connectivity; nMEANs, means of normalized FC values; (n)CVs, coefficients of variance of (normalized) FC values.

## Appendix

Considering two signals *X*_*1*_ and *X*_*2*_, short-time Fourier transform produces two complex values *F*(*X*_1_) and *F*(*X*_2_) in the following equations,

$$F\,\left(X_{1}\right)=A_{X_{1}\,\left(t,f,k\right)}\cdot \exp\left(i\,\theta_{X_{1}\,\left(t,f,k\right)}\right)$$

$$F\,\left(X_{2}\right)=A_{X_{2}\,\left(t,f,k\right)}\cdot \exp\left(i\,\theta_{X_{2}\,\left(t,f,k\right)}\right)$$

where, *t*, *f* and *k* represent indices of time, frequency and trials, respectively. Event-related phase-locking value *PLV* (Lachaux et al., 1999) and coherence *ERCoh* (Andrew and Pfurtscheller, 1996; Lachaux et al., 2002) are computed as follows;

$$P\,L\,V_{\left(t,f\right)}=\frac{\left|\sum_{k}\exp{\left(i\theta_{X_{1}\,\left(t,f,k\right)}\right)}^{*}\bar{\exp\left(i\,\theta_{X_{2}\,\left(t,f,k\right)}\right)}\right|}{N}$$

$$\begin{array}{c} E\,R\,C\,o\,h_{\left(t,f\right)}=\frac{\left|\left(\sum_{k}F\,{\left(X_{1}\right)}^{*}\,\bar{F\,\left(X_{2}\right)}\right)\right|}{\sqrt{\sum_{k}F\,{\left(X_{1}\right)}^{*}\,\bar{F\,\left(X_{1}\right)}}\,\sqrt{\sum_{k}F\,{\left(X_{2}\right)}^{*}\,\bar{F\,\left(X_{2}\right)}}}\\ =\frac{\left|\sum_{k}A_{X_{1}\,\left(t,f,k\right)}A_{X_{2}\,\left(t,f,k\right)}\cdot \exp{\left(i\theta_{X_{1}\,\left(t,f,k\right)}\right)}^{*}\bar{\exp\left(i\,\theta_{X_{2}\,\left(t,f,k\right)}\right)}\right|}{\sqrt{\sum_{k}A_{X_{1}\,\left(t,f,k\right)}^{2}}\,\sqrt{\sum_{k}A_{X_{2}\,\left(t,f,k\right)}^{2}}} \end{array}$$

where, *N* represents total number of trials. As these equations indicate, amplitudes work as a weighting factor of cross-periodograms in computing coherence (and coherency). Focusing on a difference between these equations, we can define amplitude coherence *AC* as follows (Srinath and Ray, 2014);

$$A\,C_{\left(t,f\right)}=\frac{\left|\sum_{k}A_{X_{1}\,\left(t,f,k\right)}\,A_{X_{2}\,\left(t,f,k\right)}\right|}{\sqrt{\sum_{k}A_{X_{1}\,\left(t,f,k\right)}^{2}}\,\sqrt{\sum_{k}A_{X_{2}\,\left(t,f,k\right)}^{2}}}$$
