# Supplementary figures and images for: Comparison of Phase Synchronization Measures for Identifying Stimulus-Induced Functional Connectivity in Human Magnetoencephalographic and Simulated Data
Source: Front Neurosci. 2020 Jun 19;14:648. doi: 10.3389/fnins.2020.00648 (PMC7318889; doi:10.3389/fnins.2020.00648)

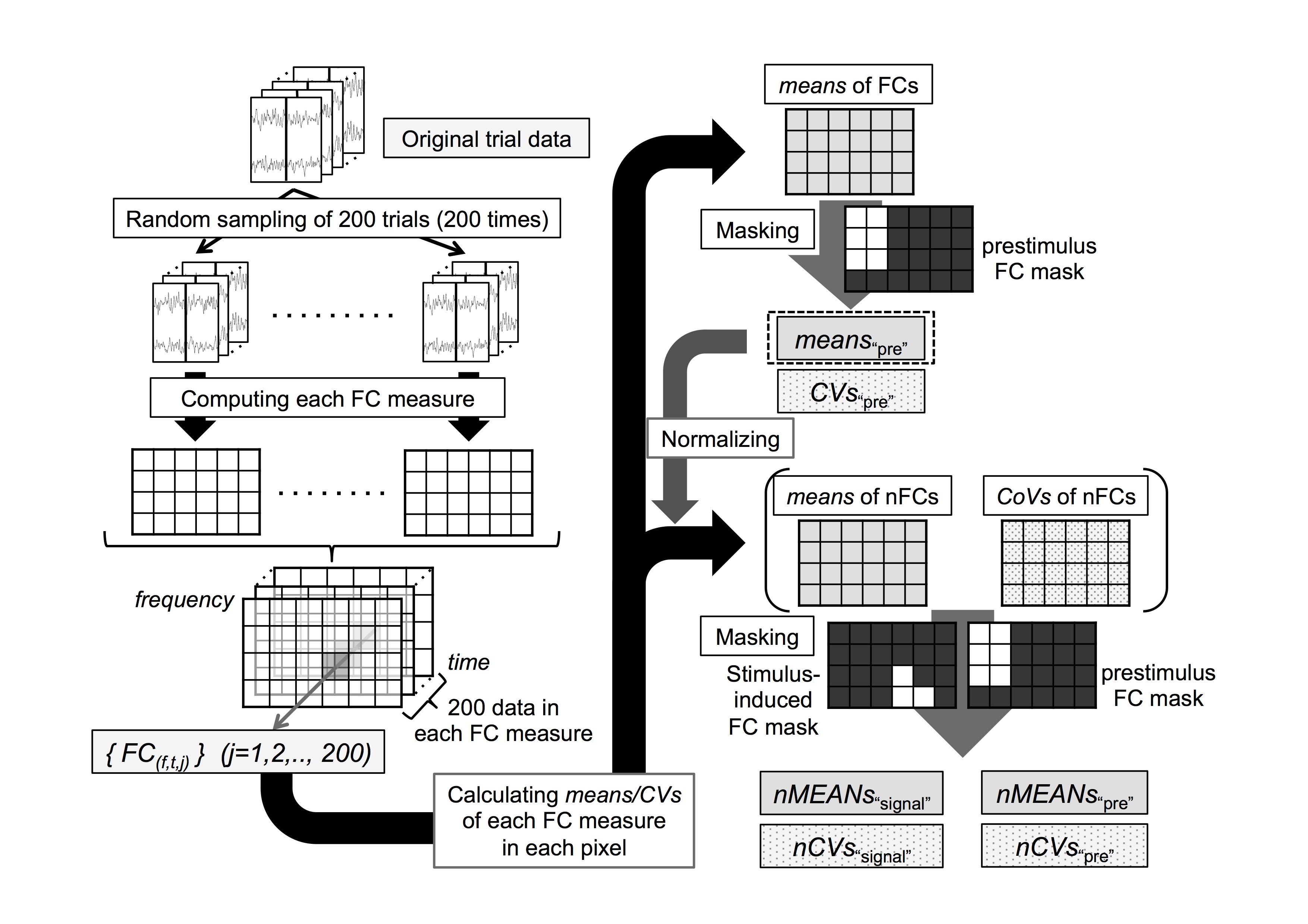

Supplement: FIGURE S1 — Processing pipeline for investigating descriptive statistics of the functional connectivity measures. The mask images for stimulus-induced FCs and for prestimulus FCs were defined using the grand-averages of double-thresholded FCs (dFCs). FC, functional connectivity; nMEANs, means of normalized FC values; (n)CVs, coefficients of variance of (normalized) FC values. [file Image_1.jpeg]
