# Supplementary material for: Comparison of Phase Synchronization Measures for Identifying Stimulus-Induced Functional Connectivity in Human Magnetoencephalographic and Simulated Data
Source: Front Neurosci. 2020 Jun 19;14:648. doi: 10.3389/fnins.2020.00648 (PMC7318889; doi:10.3389/fnins.2020.00648)
Supplement: Supplementary file 2 [file Table_1.docx]

**Supplementary material 1**

This document describes detailed contents in processing pipeline for the descriptive statistics analysis of functional connectivity measures (**Material and Methods 4.8** of the main manuscript). We conducted this analysis only for the amplitude-dependent FC measures (i.e., ImCoh and wPLI).

We computed means and coefficients of variance (CVs) of FC values from each pixel of the time-frequency space in each dataset, using the following bootstrap method (**Supplementary figure 1**). We first repeated random sampling of 200 trials from the whole trials of the original trial data (200 times). Both ImCoh and wPLI were then computed in each of these 200 random-sampled trial data (short-time Fourier transform with 333.3 ms Hanning windows with 97% overlap). To collect stimulus-induced FC values and prestimulus (or resting-state) FC values separately, we defined pixels for stimulus-induced FCs and pixels for prestimulus FCs as follows. We averaged the grand-averages of double-thresholded FCs (in **Material and Methods 4.6.1** of the main manuscript), in which the number of trials was 200, across all the four FC measures. We defined a mask image for stimulus-induced FCs as the pixels in which the values were greater than 0.05 (arbitrarily thresholding). We also defined a mask image for prestimulus FCs as the pixels ranging from 20–40 Hz in the frequency bands and 0.5–0.3 s before the stimulus onset in the time bins. We then extracted sets of 200 FC values from these two mask images.

To compare these values between ImCoh and wPLI in the same scale, we rescaled FC values from these two masks by dividing them by the averaged FC value derived from the pixels within the prestimulus FC mask in each of the FC measures (named normalized FC values). We computed means and CVs of these normalized FC values in each of the pixels within the stimulus-induced FC mask and prestimulus FC mask. We collected the means and CVs of the normalized FCs from all the datasets and compared these values between ImCoh and wPLI.
