# Supplementary material for: Comparison of Phase Synchronization Measures for Identifying Stimulus-Induced Functional Connectivity in Human Magnetoencephalographic and Simulated Data
Source: Front Neurosci. 2020 Jun 19;14:648. doi: 10.3389/fnins.2020.00648 (PMC7318889; doi:10.3389/fnins.2020.00648)
Supplement: Supplementary file 3 [file Table_2.docx]

**Supplementary material 2**

This document describes detailed contents of the data simulation mentioned in **Material and Methods 4.9** of the main manuscript.

**1. Single-pair data simulation**

We conducted data simulation by modeling noise-contaminated auto- and cross-periodograms according to the following equations.

$${X_{1}org}_{k}=\gamma\cdot exp\left( i\cdot\theta_{k} \right)\cdot\left| \mu_{k} \right|+\varepsilon_{1,k}\cdot exp\left( i\cdot\varphi_{1,k} \right)$$

$${X_{2}org}_{k}=\gamma\cdot exp\left( i\cdot\theta_{k}+\delta\right)\cdot\left| \nu_{k} \right|+\varepsilon_{2,k}\cdot exp\left( i\cdot\varphi_{2,k} \right)$$

$${C_{X}org}_{k}={X_{2}org}_{k}*\bar{{X_{1}org}_{k}}$$

$${A_{1}org}_{k}={X_{1}org}_{k}*\bar{{X_{1}org}_{k}}$$

$${A_{2}org}_{k}={X_{2}org}_{k}*\bar{{X_{2}org}_{k}}$$

where, ${X_{1}org}_{k}$ and ${X_{2}org}_{k}$ represent time-frequency transformed complex values in which the first terms represent phase-synchronized signals and the second ones represent noises in the $k$-th trial $\left( 1\leq k\leq N \right)$. $N$ is the total number of trials ($N\in\left\{ 25,50,75,100,125,150,175,200,225,250 \right\}$). The amplitudes $\mu_{k}$ and $\nu_{k}$ are random variables sampled from the standard normal distribution. The amplitudes $\varepsilon_{1,k}$ and $\varepsilon_{2,k}$ are uniformly distributed random variables on the intervals $\left[ 0.5, 1.5 \right]$. The phases $\theta_{k}$, $\varphi_{1,k}$ and $\varphi_{2,k}$ are uniformly distributed random variables on the intervals $\left[ -\pi, \pi\right]$. $\gamma$ is a parameter for controlling the signal-noise ratios ($\gamma\in\left\{ 0.1,0.15,0.02,0.25,0.3,0.35,0.4,0.45,0.5,0.55,0.6,0.7,0.8,0.9,1.0,1.2,1.5 \right\}$), and $\delta$ is the angles of phase locking between these two signals (phase lag) ($\delta\in\left\{ \pi/{12},\pi/6,\pi/4,\pi/3,{5\pi}/{12},\pi/2 \right\}$). ${C_{X}org}_{k}$, ${A_{1}org}_{k}$ and ${A_{2}org}_{k}$ are cross-periodogram and auto-periodograms of ${X_{1}org}_{k}$ and ${X_{2}org}_{k}$. Using these simulated data, we computed the four FC measures (named original FC values). Repeating this procedure 1,000 times, we obtained 1,000 values of each FC measure in each pair of the parameters (i.e., $N$, $\gamma$ and $\delta$). Signal-noise ratios $SNR$ were computed in each of the parameters $\gamma$ as follows.

$$SNR=\frac{\sum_{k=1}^{N} \left\{ \left( \gamma\cdot\left| \mu_{k} \right| \right)^{2}+\left( \gamma\cdot\left| \nu_{k} \right| \right)^{2} \right\}}{\sum_{k=1}^{N} \left( {\varepsilon_{1,k}}^{2}+{\varepsilon_{2,k}}^{2} \right)}$$

For statistical analysis, we used the following surrogate data method. In essence, we used the following data as the surrogate data.

$${X_{1}surr}_{k}=\gamma\cdot exp\left( i\cdot{\theta'}_{1,k} \right)\cdot\left| {\mu'}_{k} \right|+{\varepsilon'}_{1,k}\cdot exp\left( i\cdot{\varphi'}_{1,k} \right)$$

$${X_{2}surr}_{k}=\gamma\cdot exp\left( i\cdot{\theta'}_{2,k} \right)\cdot\left| {\nu'}_{k} \right|+{\varepsilon'}_{2,k}\cdot exp\left( i\cdot{\varphi'}_{2,k} \right)$$

$${C_{X}surr}_{k}={X_{2}surr}_{k}*\bar{{X_{1}surr}_{k}}$$

$${A_{1}surr}_{k}={X_{1}surr}_{k}*\bar{{X_{1}surr}_{k}}$$

$${A_{2}surr}_{k}={X_{2}surr}_{k}*\bar{{X_{2}surr}_{k}}$$

where, ${X_{1}surr}_{k}$ and ${X_{2}surr}_{k}$ represent time-frequency transformed complex values which have the same statistical properties except the phase relationship between signals $\left( 1\leq k\leq N \right)$. The variables ${\mu'}_{k}$, ${\nu'}_{k}$, ${\varepsilon'}_{1,k}$ and ${\varepsilon'}_{2,k}$ are sampled from the same distributions to those of $\mu_{k}$, $\nu_{k}$, $\varepsilon_{1,k}$ and $\varepsilon_{2,k}$. The variables ${\theta'}_{1,k}$, ${\theta'}_{2,k}$, ${\varphi'}_{1,k}$ and ${\varphi'}_{2,k}$ are uniformly distributed random variables on the intervals $\left[ -\pi, \pi\right]$. The parameters $N$, $\gamma$ and $\delta$ are the same described above. Using ${C_{X}surr}_{k}$, ${A_{1}surr}_{k}$ and ${A_{2}surr}_{k}$, we computed the four surrogate FC measures. Repeating this procedure 10,000 times, we obtained the null-hypothesis distributions of each FC measure in each pair of the parameters. By applying the 95th percentiles of the corresponding null-hypothesis distributions as thresholds, we binarized 1,000 of the original FC values above in each FC measure. We computed true positive rates (TPRs) of each FC measure in each pair of the parameters by simply averaging these binarized FC values.

**2. Multiple-pair data simulation**

We simulated a set of auto- and cross-periodograms, because, in (time-) frequency-domain analysis, it is important to simulate multi-pixel data, not single-pixel data. We simply generated 10 pairs of ${X_{1}org}_{k}$ and ${X_{2}org}_{k}$ as the same equations above and computed 10 FC values in each FC measure. Repeating this process 1,000 times, we obtained 1,000 sets of 10 FC values in each FC measure. For statistical analysis, in this case, we used the same approach to the value-based thresholding in the two-step surrogate data method (see **Material and Methods 4.6.1** of the main manuscript). First, we generated 10 pairs of ${X_{1}surr}_{k}$ and ${X_{2}surr}_{k}$ and computed surrogate FC values in each pair. Second, we collected the maximum value among these surrogate FC values. Third, repeating these steps 10,000 times, we obtained the null-hypothesis distributions of the maximum surrogate FC value in each FC measure. Fourth, we applied the 95th percentile of the null-hypothesis distribution to each set of FC values as the threshold in each FC measure. Finally, we computed TPRs as the proportions of the sets in which at least one of the FC values were above the threshold. We thus obtained TPRs of the four FC measures in each pair of the parameters.

**3. Descriptive statistics analysis**

We conducted descriptive statistics analysis for the amplitude-dependent FC measures computed from the single-pair simulated data in the same procedure described in **Material and Methods 4.8**. Using the surrogate FC values, we normalized the original FC values by dividing the original FC values by the averaged surrogate FC value in each FC measure. We then computed means and coefficients of variance (CVs) of these normalized FC values in each pair of the signal-noise ratios and the phase lags with the number of trials fixed to 200.
